# Social capital and women’s narratives of homelessness and multiple exclusion in northern England

**DOI:** 10.1186/s12939-023-01846-1

**Published:** 2023-03-09

**Authors:** Joanne McGrath, Stephen Crossley, Monique Lhussier, Natalie Forster

**Affiliations:** 1 Department of Social Work, Education, and Community Wellbeing, Northumbria University, Newcastle upon Tyne, NE7 7XA UK; 2 Department of Sociology, Durham University, 32 Old Elvet, Durham, DH1 3HN UK

**Keywords:** Homelessness, Social exclusion, Women, Social capital

## Abstract

Women experiencing three or more co-occurring issues (homelessness, substance misuse, mental health) are a highly vulnerable population associated with multimorbidity. Taking women’s life stories of trajectories into social exclusion in the north of England as its focus, this paper aims to explore the complexity of social contexts in which women navigate extreme health inequalities. Of the few studies that have examined women’s experiences of homelessness through the lens of social capital, most have focused on network size, rather than the quality and influence of the relationships which precipitate or contextualise experiences of social exclusion. We utilise case studies to offer a theoretically-grounded analysis which illustrates the relationship between social capital and homelessness within this population. Our results illustrate how structural contexts, and specifically social capital accrual and social bonding processes particularly pertinent to women can act to both ameliorate and perpetuate social exclusion. We conclude by arguing that health inequalities cannot be tackled as single-issue processes but instead are multi-layered and complex.

## Introduction

Homelessness is a major global social and public health concern. Previous research recognises the substantial overlap between people experiencing a “tri-morbidity” of mental ill-health, physical ill-health, and drug and alcohol misuse associated with chronic homelessness [1–3]. The specific difficulties associated with these persistent forms of social exclusion (see [4] for review) have long been identified in the US [5], and are gaining greater attention in the UK and internationally within policy and public health discourse. Moreover, findings reveal that social exclusion is highly gendered, with experiences of interpersonal violence being particularly pertinent to women [6].

As an indicator of “extreme health inequity” interlinked with marginalisation and poverty [1], homelessness is traditionally conceptualized as detachment from social institutions and informal social networks (e.g. [7]) and is a particularly potent form of exclusion. There is a close but distinct relationship between social exclusion and terms such as poverty [8], as “being poor can lead to exclusion, but exclusion is more than just being poor, it is about participation” [9]. The term social exclusion therefore recognizes the unequal power relations which stigmatize the individual, hindering their routine participation in a community [10, 11], although there are also critiques of the term [12, 13] with Veit-Wilson, for example, suggesting that there are ‘weak’ and ‘strong’ versions of the concept, depending on the extent to which structural factors or individual behaviours respectively are considered as causes of exclusion [13].

It is well established that women are under-represented in official homelessness counts which utilise the concept of ‘literal homelessness’ i.e. sleeping rough, using hostels or other emergency accommodation [10]. Women predominantly utilize informal networks such as “sofa surfing” to avoid street homelessness [14] and tend to make less use of homelessness services, postponing entering the system until sources of informal support are depleted [15]. Of the few studies which have reported women’s experiences of sofa surfing these almost exclusively focus on youth homelessness [16, 17] or sex working [18]. Sofa surfing is typically considered by policy makers as less harmful than rough sleeping, however recent studies have reported findings of increased mortality rates amongst individuals squatting and sofa surfing in line with established health risks of other types of homelessness [19]. The health impacts of prolonged sofa surfing have therefore been largely underestimated in policy and practice. This is in addition to previous findings that women have experienced or have felt vulnerable to violence when sofa surfing [20] and in temporary accommodation such as hostels, to which women in housing crisis are often referred by local authorities [21].

Women’s homelessness is closely associated with intimate partner violence (IPV), a form of domestic abuse which describes physical, emotional or psychological violence perpetrated within a romantic relationship [22]. Experience of interpersonal violence perpetrated by men is often linked to women actively avoiding services that appear designed, and dominated by, the needs of men [23]. This “spatial and policy invisibility,” [24] has significant health consequences as by utilising informal networks, women are able to remain “hidden homeless” for longer periods of time, which is associated with greater severity of health issues and higher mortality [25]. When women with histories of chronic homelessness do present to healthcare services this is often at a late stage, with a multitude of complex health issues [26].

Despite widespread acknowledgement of the unmet health needs within this population [27, 28], homelessness in Europe is increasing [28]. Women’s experiences of homelessness remain under reported, however, and such, it is not yet fully understood how women navigate critical life events associated with chronic homelessness. Homelessness and health policy in developed countries is moving upstream, focusing on prevention and the avoidance of harm [10]. Previous studies on healthcare access in homeless populations have described negative experiences and resulting health impact [28]. The aim of the study, therefore, is to analyse how women with experience of co-occurring homelessness, substance misuse and poor mental health utilise social capital within social networks to survive “persistent precarity” [29] which may help or hinder them long term and understanding of which may facilitate more timely healthcare interventions.

### Bourdieu, social capital and health

Pierre Bourdieu is arguably the most influential sociologist of the last 50 years, and his work has been extensively used by researchers across different disciplines (e.g. [30]). Bourdieu’s best-known concepts are those of *habitus*, *capital* and *field*. These concepts are relational to each other: a person’s *habitus* will affect the forms of capital they possess and pursue (and vice versa) and the various forms of capital are only of value and use when they exist in relation to others – ‘A capital does not exist and function except in a field’ ([31]: 101 original emphasis). A field may be thought of as a structured space around which capital is organised. Acting as ‘the strategy-generating principle enabling agents to cope with unforeseen and ever-changing situations’ ([32]: 72), the *habitus* is a way of being, a way of seeing and responding to the world. A key aspect of the concept is the embodied nature of the *habitus* – how people walk, the hand gestures they use, how they eat, how they greet others, the language that they use, the accent that language is spoken in. The *habitus* has been likened to a ‘feel for the game’ ([32]: 128): the embodied ability to use past experience and knowledge to practically and physically adapt and respond to the present situation, in order to maximise ones’ advantage or secure or strengthen ones position in the ‘game’.

Bourdieu’s forms of capital have remained attractive to health researchers and these have been deployed to examine and theorise the influences – good or bad - of familial and social connections on health-related behaviours (e.g. [33]). Bourdieu ([34]: 248) viewed social capital as the:

*aggregate of the actual or potential resources which are linked to possession of a durable network of more or less institutionalized relationships of mutual acquaintance and recognition.*

Social capital was, in his view, linked to an individual’s accumulation of economic and cultural capital and was more likely to be used to maintain and reproduce unequal relations, than to ameliorate their effects. By explicitly linking the transmission, deployment, and systems of rewards to the struggle for social distinction, practices of domination, and the maintenance of class-based hierarchies Bourdieu argued that social capital contributes to the accumulation and exercise of power and the maintenance of inequality [35]. As a modulating factor of social exclusion, social capital is conceptualized as resources embedded in social relationships, particularly reciprocity, obligation, and trust [36] or larger social structures such as networks and communities [37] as well as the ability of individuals to acquire these benefits or potential resources [34].

Events associated with triggering exclusion such as domestic abuse, homelessness or the onset of financial problems can be termed “biographical network disruptions” [38]. People experiencing crisis tend to seek support from networks [39], however this can substantially alter support networks, and thus their access to social capital. Interpersonal conflict and a lack of instrumental support were found to be within the top three reasons women became homeless [40]. When norms of reciprocity cannot be met for extended periods, relationships become conflictive (e.g. [41]), ultimately leading to detachment from family networks and balanced personal relationships [42]. These events affect social capital by both reducing individuals’ participation in specific social contexts and by violating general relationship norms [42].

Individuals facing unforeseen or difficult life events may therefore find themselves in a precarious situation without the necessary resources and/or support available from networks. Roca et al. [43] found that experiencing multiple negative life events has a direct impact on the effect on chronic homelessness (the so-called “revolving door to homelessness”). High incidences of traumatic events have been reported for both homeless men and women [44]. Whilst women do not generally report more negative life events than men, the amount of social support that they have has been found to predict their vulnerability [45] and have longer lasting severe impact on mental health [46].

The established finding in the literature that women’s housing experiences intersect with their social relationships, supports the hypothesis that the influence of social capital is an important factor modulating homelessness. Previous studies have investigated social network size [40, 47, 48]. The ability of certain groups to leverage social networks has been shown to serve as a buffer against the acute end of homelessness, for example by providing temporary places to stay [49]. Family ties have been shown to be highly predictive of early experiences of homelessness [50]. Therefore, the quality as well as presence or absence of social relationships can influence housing options and contribute to both entering and exiting homelessness [48]. Studies have found that homeless women have lower social support than housed women [51].

Though it has been assumed by most studies to date that larger social networks and availability of capital equates to better chances of exiting homelessness, findings relating the negative impact of social capital on health have been growing [52]. Indeed, it has been suggested that the relationship between social capital and health is a “double edged phenomenon” ([53]: 106). Given this contested role of social networks in the lives of people who are marginalized, this research sought to develop grounded understandings about ‘turning points’ in women’s lives, and how experiences of homelessness interact, within the context of other domains of deep social exclusion.

Some studies have suggested that sofa surfers may have more access to social support networks, compared with people experiencing other forms of homelessness, like rough sleepers who more often lack social support [16]. However here we see that the basic assumption that membership of networks brings about benefits for people’s health [54] is flawed because although “every support network is a social network, the reverse is not true because we can have social networks that are not beneficial and through which social support is not provided” [55]. Indeed, the very term “social support” has been called into question, because “support connotes images of encouragement and care, suggesting that responses from network members are uniformly positive” ([56]: 222). In recognition of the fact that not all networks produce norms of trust and confidence between their members, the distinction between “bonding” and “bridging” capital refers to relations between members of a network with a similar social identity (bonding), whereas bridging capital refers to relations between people who are not alike in some socio-demographic (or social identity) sense (e.g. [57]).

The concept of negative social capital [37] explains the notion that networks can have a wide range of social responses, including negative ones [58, 59]. At least four negative consequences of social capital are identified: “exclusion of outsiders, excess claims on group members, restrictions on individual freedoms, and downward levelling norms.” In an exploration of negative social capital in America’s urban core, Wacquant ([60]: 27) states that ‘affiliative ties and bonds of obligation with friends and associates in the ghetto constitute a resource for survival and success in the informal economy, but they create impediments and obstacles when attempting to move up and into the official labour market--"ties that bind” and keep you down’. Curley [61] called these ‘draining ties’, especially if more assistance and support is given than is received or if they are with ‘people that bring one down emotionally with constant complaining or involvement in their problems’ ([61]: 237). Her study of low-income women experiencing change in their housing situation and the changes in resources that are available and useful to them is particularly relevant to this study.

A more recent systematic review of the literature around the ‘dark side of social capital’ and its negative effect on health outcomes suggested two more negative consequences of social capital in addition to those identified by Portes: behavioural contagion and cross-level interactions between social cohesion and individual characteristics. The authors of the review argue that it is: “important to understand the “dark side” of social capital to avoid the trap of presenting community solidarity, social control, and collective sanctions as the panacea to solve health related problems” ([53]: 106).

Understanding social capital is therefore crucial to explain how marginalised people experience a higher risk of falling into social exclusion [62], though it is not fully understood to what extent social capital can help or hinder. To our knowledge there is no research utilising a social capital perspective into the experiences of women whose experience of homelessness becomes chronic. Drawing on Bourdieu’s framework, feminist scholars and social theorists have critiqued and expanded his work generally, and the concept of capital to include areas such as feminine capital [63] enabling greater scope to apply Bourdieu’s ideas to illuminate the intersection of homelessness, and relationships and gender-based violence. As an alternative viewpoint to discourses that commonly categorize homeless women as engaging in risky behaviours, this offers a way to discuss critical life events whilst acknowledging the impact of structural disadvantage and social exclusion. It can therefore illuminate how women navigate the complexity of homelessness and substance misuse, which might reduce some of the negative health consequences by facilitating earlier intervention.

## Methods

### Overview of study design

The current study was part of a wider ongoing examination of the experiences of homeless women with co-occurring issues accessing services and the impact on health. During the coding process, social capital was identified as one of the prominent themes in the data, though we did not originally set out to investigate this and it emerged naturally in the narratives. Interviews were conducted between October 2021 and February 2022, in the north of England at two drop-in services for anyone homeless or vulnerably housed.

### Participants

A total of 20 women participated in one-to-one interviews lasting 25 mins to 140 mins, and of these, 16 met inclusion criteria and were included in this study. Participants self-reported their current housing situation and number of episodes of homelessness. They were also asked if they had any diagnosed or self-reported mental or physical health problems, which were coded as yes or no responses. Finally, demographic questions included age, and services used in the past 12 months.

Participants were either identified and invited directly by staff or recruited via opportunity sampling. Convenience and snowball sampling methods were used to recruit further participants, and efforts were made to recruit women not currently engaging with services. Staff at each location facilitated in-person introductions to the researcher, who briefly introduced the study, and then screened potential participants for inclusion. One participant was rough sleeping and was recruited directly from the street. Participants were interviewed at each location until data saturation was reached.

Inclusion criteria was met if individuals identify as women, were over 18, single and were currently homeless and / or had experienced more than 3 separate episodes of homelessness (homelessness was defined as, rough sleeping, staying in a hostel or sofa surfing, see Table 1); had experience of either current or previous chronic substance misuse; and either diagnosed or self-reported mental health issues.

**Table 1:** Episodes of homelessness: (*n* = 16)

	0	1	2	3+
Episodes of rough sleeping	38%	6%	6%	50%
Episodes of hostel stays	31%	25%	25%	19%
Episodes sofa surfing	-	6%	13%	81%

### Demographics

Participants ranged in age from 25 to 58 (mean age 41). Their current living situation was reported as either currently homeless (38%), supported housing (50%), private rented accommodation (6%) and 6% were vulnerably housed in unsuitable accommodation.

Primary presenting substance misuse was reported as opiates (74%), alcohol (13%), and benzodiazepines (13%). Self-identified poor mental health was characterised as suffering from long term depression and anxiety, and 100% had trouble sleeping. 63% of participants reported suicidal ideation within the past 12 months. 25% reported that they had ever been admitted to a psychiatric facility. A minority had psychiatric diagnoses (6%) which were reported as schizophrenia and personality disorder.

Within the previous 12 months, 56% had accessed housing support services, 56% had used substance misuse services, 38% had accessed support for domestic abuse and 25% had accessed mental health support. Fifty percent had visited their GP at least once. Significant acute healthcare use was reported, with at least 18 individual admissions to A&E reported within the group in the previous 12 months.

### Procedure

Participants were taking part in a wider study and responded to three measures during one-on-one interviews, beginning with demographic questions, and followed by the Multidimensional Scale of Perceived Social Support (MSPSS) and semi-structured interview. A timeline approach was utilized by the researcher, which helped to organize the data and provided a participative space in which to discuss sensitive topics. In some cases, the participants spontaneously started talking about their lives and in this case the researcher allowed them to talk freely without interruption and reordered the interview as seemed to fit best, whilst ensuring that the basic interview topics were covered. In this paper we report on the qualitative elements of the study.

### Ethical considerations

The lead researcher carefully explained the scope and purpose of the study to all participants. All were offered the opportunity to use a pseudonym of their choice. All quotes and characteristics which might identify participants are anonymized. All data were self-reported. Audio recordings and all written documentation of research data were stored on a password protected server, only accessible to the research group. All women were offered the chance to meet with a support worker immediately following the interview. If any signs of distress were observed, the interview was stopped and the lead researcher checked how they were feeling and whether they wanted to continue, reminding them that they were under no obligation to continue and could stop at any time. Interviews were paused on two occasions, however both participants indicated that they wanted to continue. Afterwards, none of the women took up the offer of a debrief, however all said they had found taking part in the research a positive experience.

### Analysis

All interviews were audio-recorded and afterwards transcribed by the lead researcher to increase familiarity with the data. After checking the transcripts for transcription errors, meaning was constructed into the data using a grounded theory approach. Transcripts were explored by the lead researcher for pertinent themes and discussed with the research team.The data were labelled line by line using open coding as we asked, “what is this an example of?” Open coding was used to identify basic themes relating to women’s experiences (see Table 2) and decision-making processes in relation to homelessness across the 20 interviews. Concepts such as trauma, interpersonal violence, and child removal. Were grouped under category labels. Each category was considered in terms of its characteristics and as differences and similarities emerged, we collapsed our initial collection of concepts into a code list of important concepts. It was at this stage that the centrality of networks and resources, and thus social capital, in the discussion became apparent. Interview transcripts were then re-examined to identify broader themes (or forms) of agency under which certain basic themes could be grouped.

**Table 2:** Number of participants who identified different experiences (*n* = 16)

	identified	disagreed	not addressed
*Habitus* of instability			
Early trauma	88% (14/16)		12% (2/16)
Care experienced	69% (11/16)	31% (5/16)	
Homeless before age 21	63% (10/16)	31% (5/16)	6% (1/16)
Hidden homelessness			
Significant trauma whilst homeless	56% (9/16)		44% (7/16)
Domestic abuse			
Experienced physical abuse	88% (14/16)	6% (1/16)	6% (1/16)
Experienced emotional abuse	88% (14/16)		12% (2/16)

### Findings

#### *Habitus* of instability: anticipating crisis

Fragmented, often abusive early settings characterised by poor relationships with one or both parents characterised participants sense of their place in the world. The family home was frequently associated with experiences of physical, emotional and sexual abuse. With the disruption of formative networks and bonds with caregivers, this culminated for many within institutional care or the care of relatives. Reported experiences of care were mixed, with many women describing “getting in with the wrong crowd” and taking drugs for the first time but also feelings of relief during a respite from abuse at home:

*Me mam was a severe alcoholic. I used to get beat up daily. The school didn’t do anything until I was 12-year old, after me nanna died. And basically, I got put with the person who was actually raping me. So I was there for 3 months and the trauma of that, I just couldn’t cope with. So I rebelled at school, and that’s when I got put into […] children’s home. Things started to calm down a little bit there, but I just wanted to be – it sounds stupid – but I wanted to be where my safety net was, where my mam was* (Rosie).

Women described the home environment being one where substance misuse and interpersonal conflict were normalized. Trauma was widely experienced, with multiple adverse experiences throughout the life course. Leaving home often occurred as a result of crisis, either the death of a main caregiver or family breakdown. Women described getting into relationships with older men, which provided both a means of escape and in many cases a trap. For Michelle, a relationship initially provided a refuge from her homelife and though the relationship quickly turned sour her mother did not allow her to return home: “*I moved out when I was 15 year old I rang me mam crying cos I was miles away from [home …] and she went “you’ve made your bed you lie in it”* (Michelle).

Early experiences of abusive family life set future expectations of relationships, where physical violence was normalized and associated with love. Tracy described how unremarkable experiences of violence were, which foreshadowed later relationships:

*I was beaten as a child by my father. My mother beat my sister. Never ever hit me. Sides get picked, you get her I get her. And I thought it was how someone showed that they loved you, you know? … I had my nose broken. First my dad. And then boyfriends. There was a competition going on. It becomes a way of life I guess* (Tracy).

Early experiences of lack of informal support of parents and extended family; resources that are normative and critical to healthy child development and achievement even into early adulthood [64] impact these women throughout their lives. Experiencing early trauma, including emotional, physical, and sexual abuse, neglect, parental mental ill-health and/or substance abuse, are all particular risk factors associated with unresolved trauma and long-term homelessness in adulthood [65].

### Sofa surfing: utilising capital to navigate hidden homelessness

Typically, women had repeatedly experienced several different types of homelessness. But sofa surfing was by far the most long lasting and repeatedly found. Women had wide networks which enabled them to sofa surf for considerable periods of time without exhausting their options. This was often linked with substance misuse and needing access to drugs “*I was just sofa surfing. From drug house to drug house to drug house. Me drug addiction got worse and I looked terrible. And I met me baby’s dad through drugs… Met him quite early didn’t fancy him but it was just the drugs, I was there for the drugs* (Gillian).

Underlining the importance of understanding sofa surfing as a gateway to exclusion many women detailed the inherent danger and vulnerability in needing to rely on others to find a place to stay:

*You get to know people the wrong way sometimes. It’s really sad when you need, you know you’re doing a very dangerous thing... it also exposes the anger. Men who hate women. I always forget the word, misogynist. You become a needy woman, you meet a misogynist* (Tracy).

Survival sex is understood as “the exchange of sex for material support” [20], and has previously found to be common amongst young homeless women [66]. Moore’s study, in Australia noted that issues of consent and coercion were obscured within this context [66]. The expectation of sex was hinted at by several women and explicitly expressed by Tracy:

I was couch surfing but there was many a night where I’d have to get out of there because they assume that means sex in bed and rock and roll, you know ... Because you owe something. And once you owe something, they can take anything. It’s dirty. It’s a really ugly, you know the word rape is um, is so misunderstood even as a victim of it because if you’re doing it for a place to stay, am I being raped? Or am I f****** him so I can have somewhere to sleep. You know what I mean? Excuse my language. It’s a horrendous place to be.

The routine violence and victimisation associated with sofa surfing is less well evidenced in the literature but well known by the women in this study. In particular sexual exploitation was understood to be prevalent: “I was thinking, “Shall I mention it [sexual exploitation] and I thought well I’m not going to be the only one am I?”(Sienna). That the women in this study were still sofa surfing despite being aware of the inherent dangers highlights the paucity of other options available to them.

### The field of intimate relationships

Women leaving home or becoming homeless at an early age were particularly vulnerable to predatory relationships with older men or romantic relationships of necessity and convenience. Prior studies found a particular association between intimate relationships as a source of increased violence and transitions into injection drug among females [67]. Consistent with these findings the women in this study frequently described how partners introduced them to drugs and were the catalyst to transition into “harder” drugs:

*met a guy who was like 29 and he gave me heroin, like I’d never touched drugs in my life, he injected me, with heroin. I know. I had crack. I didn’t really know what I was taking. He introduced me to heroin at 15* (Gillian).

Intimate relationships were often based on the need for protection: “*[partner’s name] is there all the time cos she protects us. She makes us like get up in the mornings and try and get ready and get washed, and stuff like that and she’ll like cooks for us and like she is there. If she hadn’t have been, I wouldn’t be here”* (Carina).

Several women met their partners through drug networks and were subsequently coerced into drug dealing: *[I met him] through different people in […], who were selling drugs and people knowing people and me being young he swept us off my feet, wined and dined us and I was a lot younger and – he used us basically, to his advantage. “Well if you do this, do that, I’ve got me third strike I’m out, you’ll have to sell this and that”* (Delia).

Gillian illustrates the difficulty of getting away from the pull of street culture. Having had a relatively happy and stable relationship for several years, coinciding with better mental health, a job and sense of purpose, the end of that relationship swiftly led to her going back to where she felt a “connection”, a drug dealer who knew her which she associated with having a sense of belonging:

*When I was with [partner] and we were both PT [Personal Trainers] everything was fine … my life was absolutely the best … No domestic violence or nothing. I was just happy* … I *was drug free, like totally, just normal just normal shit and he broke my heart and that and I ended up, like not back on heroin just crack. I started using cocaine and drinking and that you know, cos he left us. And then from then I went out like a coke dealer who I knew from all them years ago when I had [...] who used to serve us up. And I felt like a connection with him cos he knew us* (Gillian).

Intimate relationships were entered into out of necessity, following crisis points (homelessness, death of previous partner) and women described partners exacerbating drug use, or precipitating further trauma and abuse.

### Domestic abuse: “ties that bind”

Repeated experiences of domestic abuse were apparent in the biographies of almost all women though it was not always perceived as such. Relationships were often idealised in the first few months then quickly descended into abuse:

*You think you find the right person, you think they’re so nice and everything’s perfect for the first 6 to 12 months and then after 12 months it just goes pfffft. Like woah. And by the time that’s happened you’re just too far involved. And then you end up the one that’s out on the street* (Rosa).

One of the most harmful aspects of domestic abuse is detachment from social networks, thus further deepening exclusion. Here, Sally describes being isolated her from family and friends and eventually her children: *Nobody knew what was going on. So I eventually left, and unknown to me … I was made out to be the bad person, like a complete weirdo* (Sally).

Several women described long term physical and mental health impact resulting from injuries caused by their partner. Dee was using heroin to manage chronic pain caused by physical injuries as well as trauma from abuse: “*I was married once. And I’d never do it again. He was a woman batterer. Steel plate in my head. He was so violent*” (Dee).

Other women described how their partner provided resources but also perpetuated further trauma:

*he used to say “you’ve got nobody. You’ll never go hungry if you stay with me...” And it’s just hard like. I struggle every day. So it’s like I’m either, it’s easier for food, I’d get lifts if I needed to go to places or I’m not being with that person and struggle. Erm, but not arguing and not fighting. It’s just hard* (Sienna).

Michelle describes how her relationship commands a lot of her attention and energy, with expressions of affection interspersed with mental turmoil and uncertainty:

*Me partner who lives with me, [name], he’s really well known here. He got kicked out of a hostel a while ago and that’s how I met him... he’s playing us [me] along saying he loves me and wants to be with me, and it’s ripping me to bits, my head’s battered. … he doesn’t have a good word for us. Constantly puts us down. I don’t know. But he walked away a couple of month ago when he got paid, spent £750 left me with not a penny and went away for a week and come back when he had nothing. I knew then, he didn’t love me. No-one who loved someone would do that to them. You know. I couldn’t see the lad on the streets, I just couldn’t* (Michelle).

Amongst the women who had exited homelessness, many chose to live alone: “*I mean I just don’t intend getting into a relationship to discover how to have one. I’m done. I’ve had enough bad ones. I’ve loved, and I’ve been loved back a couple of times. But it hurts even harder when they’re the ones that try to kill you”* (Tracy).

Most of the women who had successfully exited homelessness actively avoided situations where they might meet a new partner and expressed no desire for intimate relationships. This perhaps relates to not only their overwhelmingly bad experiences of relationships, but provides context to their perception of relationships primarily driven by necessity to obtain shelter, protection and resources.

### Services: a source of bonding or bridging capital?

Most of the women in our sample had stayed in a hostel at some point, as a response to crisis rather than a choice. Drug use and sharing was prevalent within hostels. The need to reciprocate and provide something in exchange for a place to stay was a factor in perpetuating drug and alcohol dependency. Some women described meeting new partners in hostels, although these were often described as abusive relationships:

*I went into the hostel... it wasn’t actually too bad, it was alright but I met somebody and I ended up, fell into a relationship with him. I fell pregnant with him and I lost the child. Cos he literally beat us, beat us up that badly that I lost the baby, so. It’s just been so many bad things* (Rosa).

Women’s reliance on social support and lack of protection under the law is highlighted by the case of women who are evicted from hostels and temporary accommodation due to their partners (and in some cases ex-partners) behaviour. People staying in temporary accommodation can be evicted without a court order, and whilst UK states that “reasonable notice” should be given before evicting people, this is not well defined in law and in practice vulnerable women are often asked to leave with very little or no notice. Claire was asked to leave a mixed-sex hostel because her ex-partner came and caused trouble: *I moved to the […] hostel but because me ex found out I was there they kicked us out of there*.

This was a particular issue for women accessing temporary accommodation as a victim of domestic abuse. Women leaving extremely violent partners were effectively penalized for their partner’s behaviour and were asked to leave hostel accommodation because their partner was dangerous:

*So eventually the police did help us in a way. By getting us a place in [place in the North East] it was a women’s battered place but erm they said they were gonna accept us and then because I told them what me partner was like, they said we can’t accept you for the safety of the other women…Me ma started crying she went “god I can’t believe it. What the bloody hell are you going to do now?”* (Diane).

Despite health and social care services being a potential source of bridging capital, we found that hostels are described as socially homogenous environments. There were few or no opportunities to “move on” within highly tight knit communities. In accordance with the description of social capital as “the pressure on an individual actor to incur costs by virtue of membership in social networks or other social structures” [68] women were subject to a wide range of harmful obligations, including partaking in substance misuse, sex for rent and criminal activities.

## Discussion

Key findings in this study lend support to the idea that social capital is a “double edged phenomenon” which impacts on health and housing vulnerability [53]: 106. We aimed to examine how women with co-occurring homelessness, substance misuse and poor mental health utilise social capital and to what extent it is helpful or hindering to them. Lack of social capital has long been recognised as a risk factor to enter homelessness at a young age (e.g. [50]), and our findings add to this by suggesting how social capital influences the occurrence of long term homelessness. In this context, a more nuanced scenario emerges that reveals how ‘[d]ifferent material conditions offer different possibilities for value accrual’ ([69]: 509). Social capital theory may offer an alternative to individualizing narratives often associated with homeless women, focusing on “success” or lack of, which does not recognize how marginalized women may simply be drawing upon the (limited) resources that are available to them [70].

Previous studies have described the phenomenon whereby bonding capital can be both an important survival mechanism in adverse contexts and also a health liability [71]. The women in this study utilised the social capital at their disposal to avoid rough sleeping but this was also negative and binding, creating obligations instead of leverage. In accordance with previous findings that whilst providing support, social relationships also serve as conduits for values, norms, social pressure, and control [42], one of the findings of this study is the extent to which interpersonal relationships can play a role in shaming, stigma and exploitation for women (e.g. [72]).

Women’s narratives in this study were highly influenced by experiences of interpersonal relationships. Early family relationships tended to be chaotic and abusive. Entry into homelessness was associated with leaving the family home and forming intimate relationships quickly established out of necessity to avoid rough sleeping. Once their dependence on their partner was established, whether for love and affection, access to drugs, or housing, they often quickly became abusive and exploitative. One of the few studies to examine women’s support networks found that a “small but definite” portion also abused them, caused trauma, and facilitated their drug use [73]. Experience of physical and emotional abuse was common, and linked with multiple traumatic experiences which was an important factor in multiple exclusion, having significant long term impact on physical and mental health.

This study adds context to preliminary findings of the long-term negative health impacts of sofa surfing [19]. The majority of women in our study described frequent periods of sofa surfing, and this included older women in their 50’s as well as the more commonly reported younger women. An unexpected finding was the lengthy periods of time that women could remain hidden homeless whilst sofa surfing, with several episodes reported up to 10 years. Sofa surfing was closely associated with sexual exploitation. Women described rebuffing sexual advances, whilst noting on other occasions that consent was not explicit. To this extent, interpersonal relationships, even those that involve violence could be perceived as a “viable context-specific option to manage the external violence of homelessness” [20] whilst undermining access to other resources such as stable accommodation and support from friends and family.

The difficulty in leaving abusive relationships was high for these women, who found themselves limited in several ways. Homogenous networks have been found to reproduce social inequalities [74], whereas Blokland and Noordhoff [75] noted that people experiencing poverty often used strong bonds rather than weak ties to help them meet their needs. Developing strategies and maintaining strong bonds to get through the day can often work to hold people back – the ‘downward levelling norms’ identified by Portes. This was very literally experienced by women who formed relationships to avoid homelessness but then found themselves having to hide from former partners and associates due to threats of violence.

Womens’s experiences of hostels perpetuated the homogeneity of their networks, often serving to deepen inequalities rather than ameliorate them. The women who were either evicted or excluded from temporary accommodation due to their ex-partners behaviour found that associations with previous partners were difficult to break. Some women who moved into their own tenancies, then found that ex-associates would find them. In this case they preferred to rough sleep rather than stay in a known location where they were at risk of harm.

When looking to develop more effective interventions, the lack of availability of bridging capital within this group is important because this has been shown in other studies to positively impact health [76]. This suggests an approach where services could make a positive impact. Whilst the women in our study did not actively avoid health and social care services as previously reported in the literature, the need for earlier recognition of risk factors leading to chronic exclusion was apparent. Women described their experiences of accessing services as not feeling listened to, and the prevalence of experiences of sexual exploitation underlines that women need time and a safe space to discuss sensitive and potentially upsetting topics. Many women alluded to sexual exploitation without specifically naming it, which could lead to under-reporting.

Womens’s experiences of hostels perpetuated the homogeneity of their networks, often serving to deepen inequalities rather than ameliorate them. The women who were either evicted or excluded from temporary accommodation due to their ex-partners behaviour found that associations with previous partners were difficult to break. Some women who moved into their own tenancies, then found that ex-associates would find them. In this case they preferred to rough sleep rather than stay in a known location where they were at risk of harm. Highlighting the gap where policies do not always recognise the reality of these women’s lives, leaving the property like this would lead to problems securing future tenancies as local authorities in England are not legally obliged to rehouse tenants who are found to have either abandoned the property or made themselves “intentionally homeless” as defined by national housing regulations.

This is an important finding for housing led approaches such as Housing First which aim to work with the most vulnerable and chronically homeless populations. Housing First is a low barrier approach to permanent supported housing which does not require prior lifestyle change. Our findings that social capital not only has a major impact on a person’s ability to acquire, but also successfully sustain housing suggest that consideration of social capital may therefore improve outcomes when supporting women to exit long term homelessness and this could be one area for future exploration.

### Limitations

Despite efforts to recruit a more ethnically diverse sample, all of the participants were white, cis-gender women. The majority of participants were recruited at a housing advice drop in centre, which might bias the findings towards women who were willing to access services. Efforts were made to recruit participants not engaging with services via snowball sampling and this was successful. Many of the participants were not currently homeless but were reflecting on their experiences. Though the distance from the event created space to reflect which elucidated some rich data, this could also introduce some inaccuracies to recollections. Nevertheless, our findings align with previous literature.

## Conclusion

This study is the first to investigate women’s experiences of co-occurring homelessness, substance misuse and poor mental health through a social capital lens. Our findings highlight the persistence of social exclusion within this population. Complex interactions between social capital and health often contrasted with singular and inflexible approaches of services women approached for support. This study suggests that individual-level explanations for health impacts of social exclusion should be replaced by approaches that acknowledge the impact of social and structural determinants of health. Findings can inform future research, the development of policies to address hidden homelessness and improve support services for women including earlier and more effective interventions.

## Data Availability

Anonymized datasets can be accessed on request by applying to the lead author.
